# Quantification of Stable Isotope Traces Close to Natural Enrichment in Human Plasma Metabolites Using Gas Chromatography-Mass Spectrometry

**DOI:** 10.3390/metabo8010015

**Published:** 2018-02-14

**Authors:** Lisa Krämer, Christian Jäger, Jean-Pierre Trezzi, Doris M. Jacobs, Karsten Hiller

**Affiliations:** 1 Department of Bioinformatics and Biochemistry, BRICS, Technische Universität Braunschweig, 38106 Braunschweig, Germany; lisa.kraemer@tu-bs.de; 2 Luxembourg Centre for Systems Biomedicine, Université du Luxembourg, 4362 Esch-sur-Alzette, Luxembourg; christian.jaeger@uni.lu (C.J.); jean-pierre.trezzi@uni.lu (J.-P.T.); 3 Integrated Biobank of Luxembourg, Luxembourg Institute of Health, 3555 Dudelange, Luxembourg; 4 Unilever R&D Vlaardingen, 3133 AT Vlaardingen, The Netherlands; doris.jacobs@unilever.com; 5 Helmholtz Zentrum für Infektionsforschung, 38124 Braunschweig, Germany

**Keywords:** GC-MS, stable isotope labeling, mass isotopomer distribution (MID), plasma, nutrition

## Abstract

Currently, changes in metabolic fluxes following consumption of stable isotope-enriched foods are usually limited to the analysis of postprandial kinetics of glucose. Kinetic information on a larger diversity of metabolites is often lacking, mainly due to the marginal percentage of fully isotopically enriched plant material in the administered food product, and hence, an even weaker ^13^C enrichment in downstream plasma metabolites. Therefore, we developed an analytical workflow to determine weak ^13^C enrichments of diverse plasma metabolites with conventional gas chromatography-mass spectrometry (GC-MS). The limit of quantification was increased by optimizing (1) the metabolite extraction from plasma, (2) the GC-MS measurement, and (3) most importantly, the computational data processing. We applied our workflow to study the catabolic dynamics of ^13^C-enriched wheat bread in three human subjects. For that purpose, we collected time-resolved human plasma samples at 16 timepoints after the consumption of ^13^C-labeled bread and quantified ^13^C enrichment of 12 metabolites (glucose, lactate, alanine, glycine, serine, citrate, glutamate, glutamine, valine, isoleucine, tyrosine, and threonine). Based on isotopomer specific analysis, we were able to distinguish catabolic profiles of starch and protein hydrolysis. More generally, our study highlights that conventional GC-MS equipment is sufficient to detect isotope traces below 1% if an appropriate data processing is integrated.

## 1. Introduction

In the field of nutrition, the application of high-throughput metabolomics technologies has increased remarkably over the past decade. The great potential of this technique was especially recognized for biomarker discovery and dietary intervention studies [1]. Numerous examples illustrate that postprandial metabolomics data from body fluids, such as urine, plasma, etc., contribute to a better understanding of the consequences of a dietary intervention on metabolism [2,3,4,5,6]. Metabolomics data capture a snapshot of the metabolic state of any investigated biological system by providing information on absolute or relative metabolite levels at a distinct time point [2]. These data enable a rather static view on the investigated system, but do not allow for the drawing of conclusions on metabolite turnover or metabolic fluxes [7,8]. In this context, stable isotope labeling and time-resolved sampling are effective tools to capture dynamic flux information of the system of interest [7,8].

Several studies have demonstrated the potential of stable isotope labeling to elucidate the dynamics of human central carbon metabolism [7,9,10,11]. To specifically determine the kinetics of glucose turnover as a response to a nutritional intervention, sophisticated methods have been developed. The application of dual label stable isotope techniques, for which subjects receive a primed-continuous infusion of a stable isotope tracer (d-[6,6-^2^H_2_]glucose solution) and consume a second tracer orally (e.g., ^13^C-labeled plant-based food product) followed by time-resolved blood sampling, is a common method applied in nutritional research [12,13,14,15]. For some applications, this methodology can further be extended by a third tracer to enhance the accuracy of rate determination [15,16]. However, all these methods have two major limitations: first, they are focused on glucose only and do not provide kinetic information on other plasma metabolites. Although glucose is probably the most important metabolite to look at, the kinetic parameters of other postprandial plasma metabolites, such as the glucose-derived metabolite lactate, the tricarboxylic acid (TCA) cycle metabolite citrate, or amino acids like alanine or glutamate, are of similar importance. Dynamic information about these metabolites allows for the study of metabolic fluxes in metabolic pathways further downstream of glucose in response to an intervention. Similar analyses have already been applied to study protein utilization [17] or synthesis [18], and postprandial lipid metabolism [19]. The second limitation is the high costs and efforts to produce the required stable isotope tracers [20]. While ^13^C-labeled substrates like glucose or glutamine are readily available, the production of labeled food products, like wheat flour, is extremely intricate. U^13^C-wheat plants require the same growth conditions as their regular counterparts, except that they have to be cultivated in a saturated ^13^CO_2_ atmosphere. As a result, all wheat flour components, such as starch (~63–72%) and wheat protein (~12%), will be fully ^13^C-enriched [7,21,22]. To keep such analyses cost-effective, the amount of tracer used in an experimental setup is kept at the required minimum, and does not normally exceed 2% of isotopically enriched food product. Although the amount of uniformly labeled (M6) glucose isotopologues is still above 1%, and thus detectable with routine GC-MS setups, enrichment in metabolites further downstream are diluted with endogenous unlabeled compounds, typically producing enrichments in the range of 0.01–1.00%.

Typically, the ^13^C enrichment is quantified in the form of mass isotopomer distributions (MIDs). However, during GC-MS measurement, not only the ^13^C labeling introduced by e.g., the consumption of isotope-enriched food products, but also naturally occurring isotopes are detected. Thus, the detected signal intensities need to be corrected to extract the ^13^C labeling of added by the tracer. In a targeted approach, as presented here, a correction matrix can be constructed based on the sum formula of the fully derivatized ion fragment [23]. The correction accounts for naturally occurring isotopes in the target fragment including the attached atoms of the derivatization agent.

Taken together, the administration of stable isotope-enriched food products, in which macronutrients like starch and protein are fully ^13^C-labeled, enclose the potential to trace kinetics of many metabolites of central carbon metabolism [21]. The major challenge, thereby, is the accurate determination of isotopic ^13^C enrichment below 1% in plasma metabolites by using standard GC-MS analysis. To address this challenge, we combine routine selected ion monitoring (SIM)-based GC-MS analysis with a specific mathematical data processing to accurately quantify ^13^C-isotopic enrichment patterns and retain information on the distribution of all mass isotopomers for ultra-low enriched metabolites. The leading advantage of our approach is that it can be performed on standard GC-single quadrupole MS instruments that are present in many labs.

For this study, we selected polar target metabolites from three substance classes, namely organic acids, amino acids, and sugars. Glycolytic activity is covered by the metabolites glucose, pyruvate, and lactate, whereas the TCA cycle is represented by citrate, α-ketoglutarate, succinate, fumarate, and malate. In addition, we further included several non-essential amino acids, such as alanine, glycine, serine, glutamate, and glutamine, and some essential amino acids, like valine, leucine, isoleucine, and tyrosine, in our workflow. We also target ketone body metabolism with 3-hydroxybutyrate, and finally, the sugar alcohol erythritol, which has recently been linked to weight gain [24].

Our analytical workflow comprises three separate stages: metabolite extraction, GC-MS measurement, and data analysis. To optimally separate the target metabolites from the plasma matrix, we decided for a liquid–liquid extraction (LLE) using a solvent/water mixture. The highest extraction yield will be obtained if non-covalent bonds between protein and metabolites break, plasma proteins are precipitated, and the target metabolites are highly soluble in the extraction fluid [25]. For this reason, we tested several LLE protocols to identify the most suitable protocol for our workflow. In the second stage, the GC-MS measurement, we aimed for a short run time to enable a high-throughput performance, and at the same time, maintain a sufficient chromatographic separation. Due to the particular importance of data processing in this workflow, we extended our MetaboliteDetector software package accordingly [26].

## 2. Results

We herein describe the optimization of the analytical procedure and a data analysis approach to generate accurate and highly reproducible mass isotopomer distributions (MIDs) for weakly ^13^C-enriched metabolites in human plasma.

The applied optimization strategy can be divided into three stages: (I) the plasma extraction method, (II) the gas chromatography, and (III) the data analysis using MetaboliteDetector [26]. We evaluated our workflow on defined dilutions of ^13^C-enriched metabolites in human plasma, and determined reproducibility, accuracy, and detection limits in this matrix. The applicability of the workflow was further demonstrated on time-resolved postprandial human plasma samples from three subjects that consumed ^13^C-enriched wheat bread (2% of U^13^C wheat flour) generated in the course of a nutritional intervention study [27].

### 2.1. Optimization of Metabolite Extraction from Plasma

**Figure 1:**
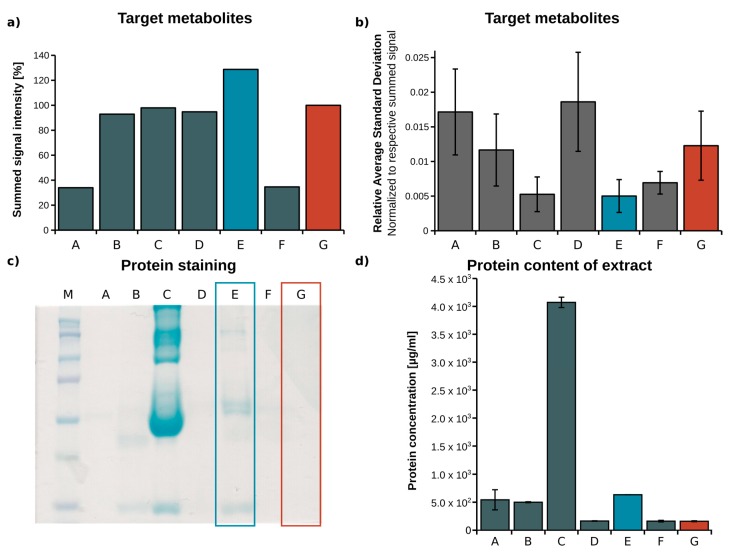
Optimization of the metabolite extraction protocol from plasma—(**a**) Summed sample signal intensity (peak area) of all target metabolites; (**b**) relative average standard deviation of all target metabolites in relation to respective summed signal; (**c**) SDS-PAGE and Coomassie staining of plasma extracts; (**d**) protein concentration of plasma extracts (A—isopropanol/acetone 1:2, B—methanol, C—acetonitrile/H_2_O, 3:1, D—H_2_O/acetonitrile/methanol 1:2:2, E—methanol/H_2_O, 5:1, F—acetonitrile, G—methanol/H_2_O, 8:1), (blue—optimized, red—reference method).

To optimize the extraction yield of all target metabolites, we tested seven different extraction protocols established for plasma samples [25,28,29]. After GC-MS measurement of the derivatized samples, signal intensities and the respective standard deviations of the target metabolites were determined (*n* = 3). The signal intensities of all target metabolites were summed, and the average standard deviation of all replicates was calculated to compare the extraction efficiency of the selected protocols (Figure 1a,b). Using protocol E (methanol/water mixture, 5:1, *v*/*v*), the summed signal intensity was increased by 25%, while the coefficient of variation over all target metabolites was decreased by 50% when compared to protocol G (methanol/water mixture, 8:1, *v*/*v*). Based on substance classes as shown in Supplementary Materials Figure S1, extraction protocol E primarily increased the extraction yield for amino acids, while the extraction yield obtained for organic acids and sugars was only slightly increased. For all substance classes, the relative average standard deviations remained small, indicating a robust extraction procedure. Compared to protocol G, protocol E has a higher water content, which leads to increased solubility of polar target metabolites and reduced non-covalent protein–metabolite binding, which results in a higher metabolite recovery [25,28]. Although extraction yields significantly increased when using protocol E instead of G, due to the increased water content, less proteins may have been precipitated in protocol E. Incomplete protein precipitation could lead to enzymatic metabolite conversion, thereby falsifying the outcome. On the other hand, when metabolite–protein binding occurs, metabolites are trapped and removed during protein precipitation [30]. To estimate whether a higher water content might cause incomplete protein precipitation, we performed an SDS-PAGE analysis of the plasma extracts, and determined the respective protein concentrations, which are depicted in Figure 1c,d. As expected, the protein concentration in extract E was about 5-fold higher (600 µg/mL) compared to the reference method G (120 µg/mL). However, protein concentrations in plasma are between 80 mg/mL and 100 mg/mL, which means that we precipitated 99.3% of all proteins with protocol E, and 99.8% with protocol G. Lowering metabolite–protein binding by increasing the water content (protocol E) is beneficial for metabolite recovery, especially for amino acid recovery when compared to protocol G, where protein precipitation is slightly more complete. Therefore, protocol E represents the best compromise, and we selected it for our analytical workflow.

### 2.2. Optimization of the Gas Chromatography Measurements

**Figure 2:**
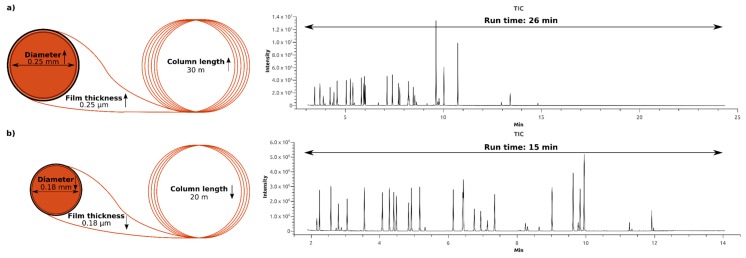
Optimization of the gas chromatography method—(**a**) GC column of 30 m in length, inner diameter of 0.25 mm and film thickness of 0.25 µm results in a run time of 26 min; (**b**) GC column of 20 m in length, inner diameter of 0.18 mm, and film thickness of 0.18 µm results in a run time of 15 min.

A gas chromatographic method suitable for high throughput is primarily characterized by a short run time. Moreover, resolution and separation of the targeted metabolites are presupposed. Applying the concept of fast GC by substituting the often (for metabolomics purposes) used column (length: 30 m, diameter, 0.25 mm, film thickness: 0.25 µm) with a shorter column with decreased diameter and film thickness (length: 20 m, diameter, 0.18 mm, film thickness: 0.18 µm) results in a shorter elution time per peak. As shown in Figure 2, the run time of the established GC method was reduced by 40%, from 26 min to 15 min. Figure 2a illustrates that the separation length of the 26 min method was not efficiently used, because all our target metabolites eluted at retention times between 3 min and 14 min. By adapting the temperature profile, the chromatographic capacity is fully utilized, and the resolution and separation of our target metabolites is improved. (Figure 2b).

### 2.3. Optimization of Data Analysis with Metabolite Detector

**Figure 3:**
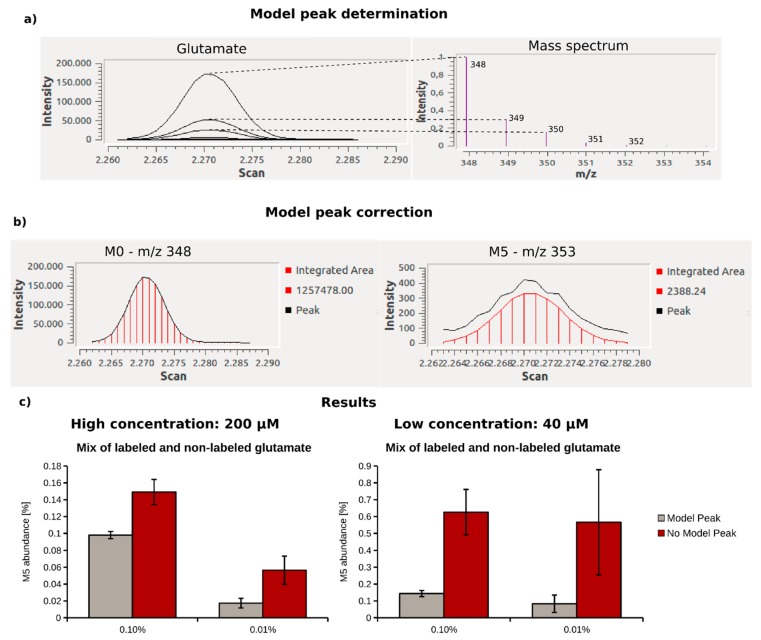
Model peak correction shown for glutamate using MetaboliteDetector—(**a**) Gas chromatographic peak and respective mass spectrum of the glutamate MSTFA derivative (Glutamate_3TMS); (**b**) the peak shape of the monoisotopic peak of glutamate (M0—*m*/*z* 348) is of high quality, and therefore used for peak shape correction of the low intensity isotopic peaks, like the 5-times labeled derivative (M5—*m*/*z* 353); (**c**) comparison of the mass isotopomer distributions (MIDs) for 0.10 and 0.01%. ^13^C-enriched glutamate at high (200 µM) and low (40 µM) concentration, calculated with (gray) and without (red) model peak correction.

When determining ^13^C enrichments below 1%, precise algorithms for the MID calculation are required. The basis of an exact MID determination is the accurate integration of peak areas (ion current/time) for all target ions. While the intensities of the most abundant, monoisotopic fragment peak are usually far above the detection limit, intensities of isotopic peaks are, in many cases, low and close to the detection limit, especially for faintly enriched compounds. Due to the superimposition with mass spectrometric noise at low intensities, the integration of such peaks can be impaired, and hinder MID determination for weakly enriched metabolites. The accuracy of peak integration can be significantly improved when mathematically fitting areas of peaks with lower abundance to the shape of a model peak obtained from peaks of the same compound, but with a higher intensity. In Figure 3a,b, this behavior is demonstrated using the mass spectrum of glutamate as an example. Since the peak shape of the monoisotopic fragment (M + 0) is identical to those of the respective isotopic peaks (M + 1–M + 5), a model peak can be constructed based on the higher quality peak shapes. This model peak is then used to correct the peak integrals of lower quality peaks. To evaluate the benefit of the applied correction, we compared MIDs obtained for different concentrations (40 µM and 200 µM), and ^13^C enrichments (0.10% and 0.01%) of glutamate using defined mixtures of non-labeled and fully labeled glutamate (U^13^C-glutamate) in aqueous solution. Data analysis was performed with and without the application of the model peak correction step (Figure 3c). Without model peak correction, we calculated an increased M5 fraction of 0.15% instead of the expected ^13^C enrichment of 0.1% at high metabolite concentration (200 µM). At low glutamate concentrations (40 µM) and for lower enrichment (0.01%), M5 abundance was drastically overestimated at 0.55%. By contrast, if the model peak correction was applied, the obtained M5 abundance matched the expected enrichments accurately at high glutamate concentrations. At low glutamate concentration and 0.10% ^13^C enrichment, the labeling was slightly overestimated, and we calculated an M5 abundance of 0.14% instead of 0.1%. At lower ^13^C enrichment (0.01%) and at low glutamate concentrations, the accuracy for MID determination further decreased. Even if model peak correction is applied, M5 abundance is highly overestimated (0.083% instead of 0.01%) when the signal of isotopic is close to the limit of detection. Although the results were still more accurate than without the application of the model peak, when both enrichment and metabolite level are low, MID determination is prone to error, and the application of our workflow reaches a limit. In conclusion, we demonstrated that the model peak correction significantly improved the accuracy of MID determination, especially when the target metabolites were low in ^13^C enrichment and/or concentration.

### 2.4. Quantification of ^13^C Enrichment in Plasma

**Figure 4:**
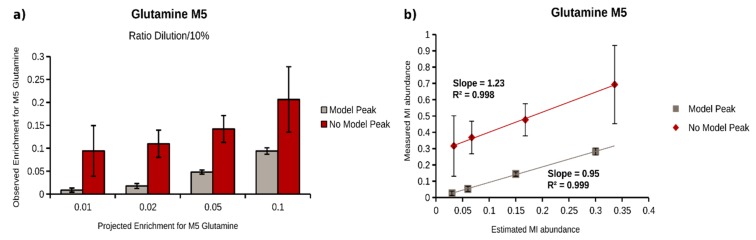
MID calculation of glutamine M5 obtained from labeled A549 cell extract diluted in plasma (0.1, 0.2, 0.5, and 1% of the original ^13^C enrichment) with (gray) and without model peak correction (red)—(**a**) Barplot of calculated (saturated) and estimated (transparent) glutamine M5 abundance with (gray) and without (red) model peak correction; (**b**) scatter plot of estimated vs measured glutamine M5 abundance, with (gray) and without (red) model peak correction for linear regression analysis.

To evaluate the potential of our workflow for plasma metabolites, we generated plasma samples with defined isotopic enrichments. For that purpose, we first incubated A549 lung cancer cells in the presence of U^13^C-glucose and -glutamine, and extracted intracellular metabolites to obtain a mixture of many labeled metabolites. We then diluted this labeled extract in (non-labeled) plasma to obtain decreasing ^13^C isotopic enrichments (1:10, 1:100, 1:200, 1:500, and 1:1000). We applied our workflow and determined MIDs for all selected target metabolites, with and without model peak correction. Out of the 25 target metabolites, 9 metabolites were included in the validation (Table 1). Based on the determined enrichments at dilution 1:10, we projected the expected ^13^C enrichment for all the other dilutions. As an example, we demonstrated our analysis for the M5 isotopologue of glutamine. The determined, as well as the projected enrichments for the dilutions 1:100, 1:200, 1:500, and 1:1000, are depicted in Figure 4. As expected, the model peak correction significantly improved the MID determination for faintly enriched M5 glutamine. With enabled model peak correction, the minimal determined enrichment for glutamine M5 was 0.026%, with an acceptable accuracy (0.002%, injections of same sample, *n* = 3) and reproducibility (0.014%, sample extraction, *n* = 3). Without this correction, we could not determine any ^13^C enrichment below 0.32% (accuracy = 0.15%, reproducibility = 0.19%). Next, we compared projected and determined fraction of M5 glutamine for all enrichments (Figure 4b), and performed a linear regression of projected and determined enrichments. The model peak correction increased the correlation between expected and observed values and, most importantly, revealed a regression line slope of 0.95 instead of 1.23, without correction. A slope of 1 would represent a perfect agreement of projected and determined enrichments. To extend these observations to further metabolites, we calculated the correlation coefficient and the slope for all ^13^C-enriched metabolites, and summarized all results in Table 1. The model peak correction increased the limit of enrichment quantification by one order of magnitude for almost all metabolites. In addition, the accuracy and reproducibility are higher, which is reflected by slopes of the regression lines closer to 1. Taken together, the application of a model peak for peak integration improves MID determination, especially for isotopologues with a low frequency, and turned out to be essential for isotopologues with fractions below 0.5%.

### 2.5. In Vivo Metabolism of Fully ^13^C-Labeled Wheat Flour

**Figure 5:**
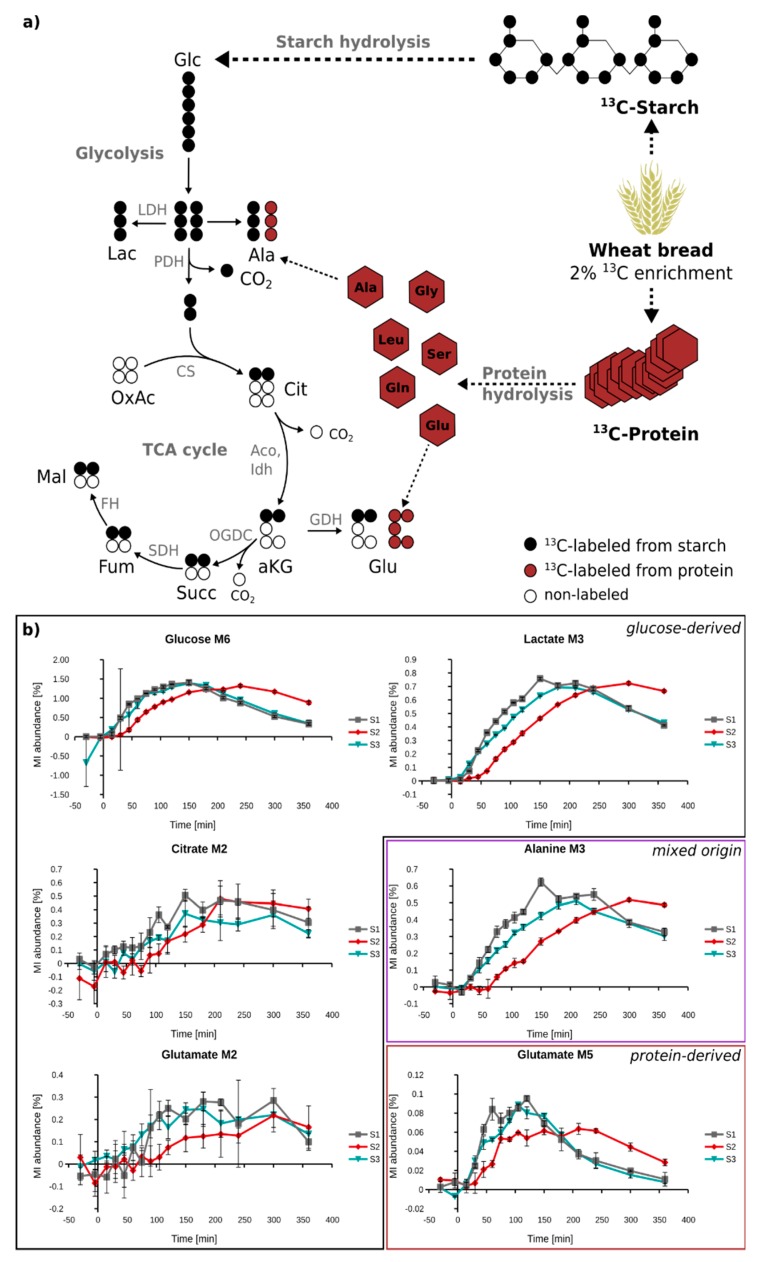
Validation of MID calculation of ultra-low ^13^C enriched plasma metabolites of subject 1 (S1), subject 2 (S2), and subject 3 (S3)—(**a**) Wheat bread with 2% of ^13^C-labeled wheat flour is composed of ^13^C-labeled starch and ^13^C-labeled protein. The labeled hydrolysis products, labeled glucose (black) and free labeled amino acids (red) enter metabolism; and (**b**) enrichment patterns of the plasma metabolites glucose (M6), lactate (M3), citrate (M2), alanine (M3), glutamate (M2) and glutamate (M5) can be measured by GC-MS. Lac—lactate, Ala—alanine, OxAc—oxaloacetate, Cit—citrate, aKG—alpha-ketoglutarate, Glu—glutamate, LD—lactate dehydrogenase, PDH—pyruvate dehydrogenase, CS—citrate synthase, Aco—aconitase, IDH—isocitrate dehydrogenase, OGDC—oxoglutarate dehydrogenase complex.

Finally, we set out to apply the enhanced workflow to study the metabolism of labeled wheat flour in human subjects. For this, we blended 2% of fully ^13^C-labeled flour with 98% unlabeled flour, and used this blend for the preparation of an Indian chapati bread. Three healthy male subjects (S1, S2, S3) then ingested this bread, and their plasma was sampled time-resolved over a period of 3 h. Already after 15 min, we started to observe M6 glucose isotopologues, reaching a maximum of 1.5% of total glucose after 150–200 min. Although the enrichment of plasma glucose was below 2%, we detected ^13^C labeling in 12 out of 25 target metabolites (Figure 5 and Supplementary Materials Figure S1), and could trace the metabolism of the wheat compounds to metabolites located further downstream of glucose. The enrichment kinetics of lactate and alanine were very similar, suggesting a close metabolic coupling of these metabolites. Interestingly, we could also observe synthesis of glutamate from wheat starch. The M2 isotopologue of this amino acid followed the kinetic of the other glucose-derived metabolites, but the isotopic enrichment was further diluted by one order of magnitude. In addition to M2 glutamate isotopologues, we observed significant fractions of M5 isotopologues of glutamate and glutamine. These isotopologues cannot be derived from glucose, and must therefore originate from wheat protein (gluten). This was also supported by the different kinetics of M5 glutamate and glutamine, compared to M2 (Figure 5 and Supplementary Materials Figure S2). Based on the appearance kinetics of just these two isotopologues, detailed information about starch and protein hydrolysis can be obtained. We observed substantial differences between starch-(M2) and protein-derived (M5) glutamate, the main difference being that protein-derived glutamate reaches its maximum earlier, compared to starch-derived glutamate. Moreover, we found clear differences in the isotope appearance kinetic of the three subjects. While the kinetics in subject 1 and 3 were very similar, all investigated metabolites were labeled later in subject 2. This observation indicates a distinct “between subject” variability in the digestion and metabolism of wheat bread. To summarize, despite the very low enrichment of 0.3% for M2 glutamate and 0.1% for M5 glutamate, we could successfully trace metabolism of wheat-derived starch and gluten down to this amino acid. Based on time resolved sampling, we observed differences between starch and protein hydrolysis. Due to the high accuracy and sensitivity of presented methodology, we could even determine between-subject variation for both processes.

## 3. Discussion

Incorporating metabolic flux analysis into metabolomics experiments expands static snapshot information on relative differences in metabolite pool sizes to dynamic information on metabolic regulation. So far, stable isotope-assisted metabolomics (SIAM) has been mainly applied to elucidate biochemical pathways in cellular systems and animals [7,31,32,33]. In human nutrition, metabolic flux analysis has, in general, been limited to the kinetics of glucose, mainly because of the high costs for stable isotope tracers. Due to the high dilution of stable isotope tracers in human metabolism in vivo, isotopic enrichment in target metabolites is very low, and in most cases, far below natural isotope enrichment. To enable SIAM in human nutrition, a robust, cost-efficient and sensitive method for the quantification of isotope incorporation is needed. Therefore, we developed the presented SIAM workflow.

A popular technology for the determination of low isotope enrichment is gas chromatography-combustion-isotope ratio mass spectrometry (GC-C-IRMS), due to its higher sensitivity, precision, and accuracy, compared to routine GC-MS [31,33]. Contrarily, the main advantages of GC-MS are the increased applicability and affordability, as well as the possibility to obtain more informative mass isotopomer distributions (MIDs), as compared to the isotope ratios of combusted metabolites obtained by GC-C-IRMS [34]. The additional value of MID data when compared to isotope ratios is demonstrated in Figure 5 for glutamate. Since this metabolite is generated either from starch or protein hydrolysis, distinct isotopologues are thereby produced (M2 vs M5), and information on starch and protein hydrolysis can be obtained by just measuring one metabolite. This information would be lost in case of GC-C-IRMS analysis. In addition, GC-MS measurements allow for higher throughput when compared to GC-C-IRMS [33], which is especially important in large-scale time-resolved dietary intervention studies with hundreds or thousands of samples.

To overcome the GC-MS related sensitivity issues, we put a strong emphasis on the optimization of the analytical method, as well as the computational data processing. Initially, we compiled a list of target metabolites involved in central carbon metabolites. The advantage of a targeted approach is obvious for this application, as the chemical properties of the metabolites are known, which facilitates the selection of applicable extraction and mass spectrometric methods [25]. For the extraction of diverse compound classes, such as sugars, organic acids, and amino acids from plasma, we suggest applying an extraction fluid composed of methanol and water in a ratio 5:1. The increased polarity compared to other mixtures (e.g., methanol/water, 8:1) reduces protein–metabolite binding, thereby promoting higher extraction yields for polar metabolites.

We applied the concept of fast GC to reduce the measurement time, while maintaining and even increasing the resolution and separation of the target metabolites [35]. According to this concept, the run time of a GC measurement can be simply reduced by using a shorter column with reduced inner diameter and film thickness. When reducing both the inner diameter and the film thickness, the plate height, H, decreases according to the equation of Golay, which results in an increase in the efficiency N and the chromatographic resolution R_s_ [35]. By using a 20 m column with an internal diameter of 0.18 mm and a film thickness of 0.18 µm, we were able to reduce the run time by 40%, while at the same time increasing the separation of our target metabolites.

Fitting low abundant isotopic peaks to the shape of higher abundant monoisotopic peaks has proven successful in GC-MS based analysis [26,36,37]. We extended this practice to increase the quality of MID determination. In our workflow, the peak shape correction increased the limit of isotopic detection and quantification by one to two orders of magnitudes (Table 1). Especially when the concentration of the target metabolite was in the lower range, MID calculation was highly improved when the model peak correction was applied (Figure 3).

Previous studies involving isotopic enrichment below 1% were mainly focused on single or a few target metabolites e.g., glucose [38], cholesterol in different species [34], or phenylalanine and leucine in muscle protein synthesis [39]. We widened this scope by targeting 25 metabolites of central carbon metabolism that are involved in different metabolic pathways, such as glycolysis, TCA cycle, and amino acid metabolism. Phosphorylated metabolites, like phosphoenolpyruvate and various pentose phosphate pathway metabolites, e.g., glucose-6-phosphate, were not taken into account, because these are not stably accumulating in blood [40]. By dissolving labeled cell extracts in human plasma, we simulated low ^13^C enrichment in plasma metabolites, and were able to quantify ^13^C-enriched isotopologues of 9 out of 25 target metabolites, with the lowest enrichment being between 0.005% (lactate) and 0.5% (fumarate), with a reproducibility between 0.001% (lactate) and 0.12% (fumarate) (Table 1). Due to the fact that we labeled the cells with U^13^C-glucose and -glutamine, not all target metabolites get labeled. For example, the essential amino acids valine, leucine, and isoleucine are not synthesized by these cells, and are thus not isotopically enriched, and could not be taken into account for the validation. For a number of metabolites, including the TCA cycle intermediate α-ketoglutarate and aspartate, no ^13^C isotopic enrichment was detected due to the combination of strongly diluted enrichment and low metabolite concentrations [41,42]. Moreover, it was not possible to reproducibly determine ^13^C isotopic enrichment for pyruvate, due to instability of the derivative [43]. For the M3 isotopologue of glucose, representing a marker for gluconeogenesis [44], the isotopic enrichment was also too strongly diluted.

Taken together, our workflow allows for measuring the enrichment of several important metabolites down to a sensitivity of 0.01%. Although we could significantly increase the overall sensitivity for MID determination, we were not able detect isotopic enrichments as low as 0.0001–0.0005%, values which have been reported for GC-C-IRMS [31,45].

To validate our workflow in a real case scenario, we applied it to analyze time-resolved plasma samples from three different subjects who consumed wheat bread enriched with 2% fully labeled wheat flour, and detected ^13^C labeling in 12 out of 25 target metabolites. We demonstrated that we can trace two distinct and independent routes of flour catabolism, starch, and protein hydrolysis. We were able to follow the dynamics of starch hydrolysis and that of further metabolites downstream of glucose. Glucose M6 starts appearing and reaching a maximum after 150 min for subject 1 and 3, and after 250 min for subject 2. Depending on the metabolic distance, the maximum ^13^C enrichment of downstream metabolites is delayed (lactate M3—170 min, alanine M3—200 min, citrate M2—210 min, glutamate M2—210 min). On the other hand, appearance of fully labeled free amino acids originating from protein (gluten) hydrolysis represented by glutamate M5 reaches maximal ^13^C enrichment already after 100 min. Similar results were obtained for M5 glutamine and M5 valine (Supplementary Figure S2). Based on this observation, we conclude that upon consumption of wheat bread, protein hydrolysis is faster than starch hydrolysis. Moreover, we observed strong “between-subject” differences in postprandial kinetics after bread ingestion, which could partly be related to the gut microbiome [46].

## 4. Materials and Methods

### 4.1. Reagents and Materials

The reference compounds (sodium pyruvate, sodium lactate, citric acid monohydrate, succinic acid, malic acid, fumaric acid, 2-hydroxybutyric acid, oxalic acid, 3-hydroxybutyric acid, glycine, l-serine, l-valine, l-threonine, l-alanine, l-aspartic acid, l-glutamine, l-glutamic acid, l-leucine, l-isoleucine, l-tyrosine, erythronate, meso-erythritol, α-ketoglutaric acid, glyceric acid) were purchased from Sigma-Aldrich, Munich, Germany. U^13^C-Ribitol (CIL Inc., Tewksbury, MA, USA), pentanedioic-d^6^-acid (C/D/N isotopes Inc., Quebec, QC, Canada), and norleucine (Sigma-Aldrich, Munich, Germany) were used as internal standards during metabolite extraction. U^13^C-Glutamate, U^13^C-glucose, and U^13^C-glutamine were purchased from CIL Inc., Tewksbury, MA, USA. For metabolite extraction, high purity HPLC (or higher) grade solvents methanol, acetone, isopropanol, and acetonitrile, and MQ water (18.2 M·cm, <3 ppb TOC) were used.

### 4.2. Plasma Samples

The blood sampling was conducted according to the guidelines of “World Medical Associations, Declaration of Helsinki—Ethical principles for medical research involving human subjects” and registered at the Comité national d’éthique de recherché (CNER) under the project number 201107/02. Plasma was produced from whole blood by centrifugation at 4 °C and 1600*g* for 10 min with deceleration set to 1. The plasma was used for plasma extraction optimization as described in section *Metabolite extraction from plasma*.

For the *^13^C-enrichment assay with labeled A549 extract*, commercial plasma IPLA-N-50mL-K_2_EDTA (Tebu-Bio, Offenbach, Germany) was used for dilution.

To evaluate the *Applicability of the workflow on plasma from a nutritional intervention study*, we obtained plasma samples collected over a 360 min time frame from three subjects that consumed wheat bread with 2% of the wheat flour substituted with fully ^13^C-labeled wheat flour [27].

### 4.3. Metabolite Extraction from Plasma

We tested seven different metabolite extraction protocols, as described in Table 2. All extraction fluids (EF) were prepared in advance, and kept at −20 °C prior to extraction. For comparability reasons, we used 20 µL of plasma for every extraction method and adapted the volume of the required EF accordingly. Mixing was performed at 4 °C and 2000 rpm for 5 min, and centrifugation was done at 4 °C and 21,000*g*.

After pipetting the indicated volume of plasma extract into the GC vials, the samples were dried overnight at −4 °C in a refrigerated vacuum concentrator (Labconco, Kansas City, MO, USA).

### 4.4. Coomassie Staining

To compare the amount of protein present in the different plasma extracts, proteins were separated by SDS-PAGE on a 10% gel (10% Mini Protean^®^ TGX™ Precast Protein Gels, 10-well, 30 µL, Bio-Rad, Munich, Germany). To reduce the influence of the solvents on protein separation, 21 µL of each extract were dried in a refrigerated vacuum concentrator, and redissolved in 27 µL 1× Laemmli buffer. A voltage of 110 V for 1 h was applied. After protein separation, the gel was washed in MQ water for 1 h. The proteins were stained with Coomassie staining solution, composed of 2% Coomassie blue, 7.5% acetic acid, and 50% ethanol for 1 h. For destaining, the gel was transferred into a destaining solution composed of 10% acetic acid and 40% methanol for 3 h, and the solution was exchanged several times.

### 4.5. Protein Quantification

Protein concentrations were determined using the Pierce BCA Protein Assay Kit (23225, Thermo Fisher Scientific, Waltham, MA, USA), according to manufacturer’s instructions.

### 4.6. ^13^C-Enrichment Assay of Glutamate

Different concentrations (low: 40 µM and high: 200 µM) and ^13^C enrichments (0.10% and 0.01%) of glutamate were adjusted by mixing 1 mM solutions of non-labeled glutamate, and fully labeled U^13^C-glutamate in water. The targeted concentrations and enrichments were extracted in accordance with extraction protocol E, and measured by GC-MS.

### 4.7. ^13^C-Enrichment Assay of ^13^C-Labeled Cell Extract

Human A549 lung cancer cells (4 × 10^6^) were seeded in a 10 cm Petri dish in D5030 medium supplemented with 25 mM U^13^C-glucose and 4 mM U^13^C-glutamine, and incubated at 37 °C and 5% CO_2_. After 28 h, intracellular metabolites were extracted according to Sapcariu et al. [47]. Briefly, cells were washed with 0.9% NaCl. Ice-cold methanol (2.7 mL) and 2.7 mL cold H_2_O (4 °C) were added to the cells to quench the metabolism. The methanol–water mixture was added into 3 mL of ice-cold chloroform. The mixture was shaken at 4 °C and 1400 rpm for 20 min, and centrifuged at 4 °C and 17,000*g* for 10 min. The upper phase containing the polar metabolites was separated and used for further analyses. To investigate low ^13^C-enrichment of plasma metabolites, 20 µL of cell extract was dried in a 1.5 mL reaction tube at 4 °C in a refrigerated vacuum centrifuge. The dried metabolites were dissolved in 20 µL of water (100%), and 180 µL of commercially available plasma (IPLA-N-50mL-K_2_EDTA) was added to dilute the ^13^C-enrichment by 1:10. By further diluting with non-labeled plasma, we obtained the dilutions of 1:100, 1:200, 1:500, and 1:1000 of the original ^13^C-enrichment. The samples were extracted according to extraction protocol E, and measured by GC-MS.

### 4.8. Metabolite Extraction of Plasma Samples from a Nutritional Intervention Study

To evaluate the applicability of the developed workflow, protocol E was applied for the extraction of the plasma samples of a nutritional intervention study [26].

### 4.9. Metabolite Derivatization and GC-MS Measurement

*Derivatization.* Online metabolite derivatization was performed using a Gerstel MPS. Dried polar metabolites were dissolved in 15 µL of 2% methoxyamine hydrochloride in pyridine at 40 °C under shaking. After 90 min, an equal volume of *N*-methyl-*N*-trimethylsilyl-trifluoroacetamide (MSTFA) was added, and held for 30 min at 40 °C under continuous shaking.

*Established GC-MS measurement.* Sample (1 µL) was injected into an SSL injector at 270 °C in splitless mode. GC-MS analysis was performed using an Agilent 7890A GC equipped with a 30 m DB-35MS + 5 m Duraguard capillary column (0.25 mm inner diameter, 0.25 µm film thickness). Helium was used as carrier gas at a flow rate of 1.0 mL/min. The GC oven temperature was held at 90 °C for 1 min, subsequently increased to 300 °C at 10 °C/min, and held at that temperature for 5 min, resulting in a total run time of 26 min per sample.

*Optimized GC-MS measurement.* Sample (1 µL) was injected into an SSL injector at 270 °C in split mode (10:1). GC-MS analysis was performed using an Agilent 7890A GC equipped with a 20 m DB-5MS capillary column (0.18 mm inner diameter, 0.18 µm film thickness) (121-3822, Agilent, Santa, Clara, CA, USA). Helium was used as carrier gas at a flow rate of 1.0 mL/min. The GC oven temperature was held at 90 °C for 0.5 min, and increased to 220 °C at a rate of 13 °C/min. Reaching a temperature of 220 °C, the rate was increased to 325 °C at a rate of 100 °C/min, and the temperature was held for 3.5 min. This temperature profile results in a run time of 15 min per sample.

*Mass Spectrometry—Tuning.* According to the supplier’s instruction, an automated tuning routine was applied every 150 injections.

*Mass Spectrometry—SCAN mode.* The GC was connected to an Agilent 5975C inert XL MSD. The transfer line temperature was set to 280 °C, and the MSD was operating under electron ionization at 70 eV. The MS source was held at 230 °C and the quadrupole at 150 °C. Full scan mass spectra were acquired from *m/z* 70 to *m/z* 800 at a scan rate of 5.2 scans/s.

*Mass Spectrometry—SIM mode.* For MID calculation, GC-MS measurements of the metabolites of interest were additionally performed in selected ion monitoring (SIM) mode. The detailed settings for each metabolite are summarized in Table 3. Correction for naturally occurring isotopes was done based on the sum formula of each derivative. Table 4 contains the settings for the internal standards used for normalization in batch quantification. For every substance class, one internal standard was selected (norleucine—amino acids, pentanedioic-acid-*d*_6_—organic acids, U^13^C-ribitol—sugars and sugar derivatives).

### 4.10. Data Processing

Deconvolution of mass spectra, peak picking, integration, and retention index calibration were performed using the MetaboliteDetector software [26]. Compounds were identified using an in-house mass spectral library. The following deconvolution settings were applied for SCAN data: peak threshold: 5; minimum peak height: 5; bins per scan: 10; deconvolution width: 5 scans; no baseline adjustment; minimum 15 peaks per spectrum; no minimum required base peak intensity. Retention index calibration was based on an C10–C40 even *n*-alkane mixture (68281, Sigma-Aldrich, Munich, Germany). For SIM data, the following setting were applied: peak threshold: 1; minimum peak height: 1; bins per scan: 10; deconvolution width: 7 scans; no baseline adjustment; minimum 2 peaks per spectrum; no minimum required base peak intensity. Retention index calibration was based on retention time. MIDs were calculated using MetaboliteDetector’s MID wizard, and relative quantification was done using the batch quantification function.

## 5. Conclusions

The application of our developed workflow enables the accurate determination of ^13^C enrichment below 1% in up to 25 plasma metabolites with conventional GC-MS instruments. By taking advantage of this powerful method, insights into the dynamics of digestive processes, like starch and protein hydrolysis, could be revealed. Moreover, conclusions on metabolic fluxes and pathway regulations can be drawn.

## Figures and Tables

**Table 1:** MID calculation obtained from labeled A549 cell extract diluted in plasma (1:1000, 1:500, 1:200, 1:100, and 1:10) of the original ^13^C enrichment) with model peak correction and without model peak correction; determination of accuracy (3 injections) and reproducibility (standard deviation, 3 extractions) of minimal MI abundance, slope and R^2^ determined by regression analysis.

Metabolite	Concentration Range (µM)	MI	Model Peak					No Model Peak				
			Min MI Abundance (%)	Accuracy (*n* = 3)	Reproducibility (*n* = 3)/Stdev	Slope	R^2^	Min MI Abundance (%)	Accuracy (*n* = 3)	Reproducibility (*n* = 3)/Stdev	Slope	R^2^
Serine	87.9–96.2	M3	0.0207	0.0263	0.0054	0.76	0.98	0.5800	0.0728	0.0400	0.27	0.81
Alanine	302.0–330.0	M3	0.0165	0.0059	0.0089	0.82	0.99	0.8950	0.0565	0.1293	0.18	0.65
Lactate	1540–1620	M3	0.0054	0.0022	0.0013	1.02	1	0.0410	0.0062	0.0031	0.97	1
Erythronate	15.0–16.0	M4	0.2317	no data	0.1545	1.01	0.99	4.7448	1.0821	0.3720	0.48	0.82
Fumarate	2.1–2.3	M4	0.4698	2.4931	0.1227	0.9	0.92	5.2324	0.3983	1.8185	0.59	0.96
Glutamine	409.8–469.3	M5	0.0260	0.0022	0.0135	0.95	1	0.3162	0.1518	0.1856	1.22	1
Glucose	6330–6631	M6	0.2337	no data	0.0167	0.6	0.98	0.8812	no data	0.0471	0.34	0.9
Glutamate	540.1–657.0	M5	0.0293	0.0015	0.0038	1	1	0.1024	0.0094	0.0095	0.98	1
Succinate	9.6–10.4	M4	0.1001	0.0831	0.0639	0.73	0.95	1.8842	0.1708	0.3157	0.3	0.95

**Table 2:** Overview of tested metabolite extraction protocols from plasma A–G, summarizing the composition of the extraction fluid (EF), the required volume, the duration of centrifugation, and the transferred volume into the GC vial.

Identifier	Extraction Fluid	Volume EF (µL)	Centrifugation (min)	Volume Vial (µL)
A [27]	Acetone/Isopropanol, 1:2	267	5	200
B [28]	Methanol (MeOH)	60	15	56
C	Acetonitrile (ACN)/H_2_O, 3:1	180	5	140
D	H_2_O/MeOH/ACN, 1:2:2	180	5	140
E	MeOH/H_2_O, 5:1	180	5	140
F	ACN	77.5	20	68.25
G [24]	MeOH/H_2_O mixture, 8:1	180	5	140

**Table 3:** Settings applied in selected ion monitoring (SIM) mode for the detection of the target metabolites.

Metabolite	Derivatization	Dwell Time (ms)	Fragment	Sum Formula	*m*/*z*
Pyruvate	1MeOX 1TMS	10	M-15	C_6_H_12_O_3_NSi	174.1
Lactate	2TMS	15	M-15	C_8_H_19_O_3_Si_2_	219.1
Alanine	2TMS	15	M-15	C_8_H_20_O_2_NSi_2_	218.1
2-Hydroxybutyrate	2TMS	15	M-15	C_9_H_21_O_3_Si_2_	233.1
Oxalate	2TMS	15	M-15	C_7_H_15_O_4_Si_2_	219.1
3-Hydroxybutyrate	2TMS	20	M-15	C_9_H_21_O_3_Si_2_	233.1
Valine	2TMS	15	M-15	C_10_H_24_O_2_NSi_2_	246.1
Leucine	2TMS	15	M-15	C_11_H_26_O_2_NSi_2_	260.2
Isoleucine	2TMS	15	M-15	C_11_H_26_O_2_NSi_2_	260.1
Glycine	3TMS	20	M-15	C_10_H_26_O_2_NSi_3_	276.1
Succinate	2TMS	15	M-15	C_9_H_19_O_4_Si_2_	247.1
Glycerate	3TMS	15	M-30	C_10_H_24_O_4_Si_3_	292.1
Fumarate	2TMS	15	M-15	C_9_H_17_O_4_Si_2_	245.1
Serine	3TMS	20	M-15	C_11_H_28_O_3_NSi_3_	306.1
Threonine	3TMS	15	M-15	C_12_H_30_O_3_NSi_3_	320.2
Malate	3TMS	15	M-15	C_12_H_27_O_5_Si_3_	335.1
Erythritol	4TMS	20	M-90	C_10_H_24_O_4_Si_4_	320.2
Aspartate	3TMS	15	M-15	C_12_H_28_O_4_NSi_3_	334.1
Erythronate	4TMS	20	M-15	C_15_H_37_O_5_Si_4_	409.2
α-Ketoglutarate	1MeOX 2TMS	10	M-15	C_11_H_22_O_5_NSi_2_	304.1
Glutamate	3TMS	15	M-15	C_13_H_30_O_4_NSi_3_	348.1
Glutamine	3TMS	15	M-15	C_13_H_31_O_3_N_2_Si_3_	347.2
Citrate	4TMS	15	M-15	C_17_H_37_O_7_Si_4_	465.2
Glucose	1MeOX 5TMS	15	M-15	C_21_H_52_O_6_NSi_5_	554.3
Tyrosine	3TMS	10	M-15	C_17_H_32_O_3_NSi_3_	382.2

**Table 4:** Settings applied in SIM mode for internal standards.

Internal Standard (IS)	Derivatization	Ions
Norleucine	2TMS	158.1, 232.1, 260.1
Pentanedioic-acid-*d*_6_	2TMS	206.1, 239.1, 267.1
U^13^C-Ribitol	2TMS	220.1, 310.1, 323.2

## Supplementary Materials

The following are available online at http://www.mdpi.com/2218-1989/8/1/15/s1, Figure S1: Plasma extraction optimization: (a,c,e) summed sample signal and (b,d,f) average standard deviation in relation to respective summed signal for the substance classes amino acids, organic acids, and sugars and sugar alcohols for tested extraction methods (A—isopropanol/acetone 1:2, B—methanol, C—acetonitrile/H_2_O, 3:1, D—H_2_O/acetonitrile/methanol 1:2:2, E—methanol: H_2_O 5:1, F—acetonitrile, G—methanol/H_2_O, 8:1) (blue—optimized, red—reference method); Figure S2: Enrichment patterns of additional plasma metabolites of S1, S2, and S3: glycine (M2) and serine (M3) are derived from either starch or protein hydrolysis; glutamine (M5), valine (M5), threonine (M4), isoleucine (M6), and tyrosine (M9) are derived from protein hydrolysis.
