# Supplementary material for: Quantification of Stable Isotope Traces Close to Natural Enrichment in Human Plasma Metabolites Using Gas Chromatography-Mass Spectrometry
Source: Metabolites. 2018 Feb 14;8(1):15. doi: 10.3390/metabo8010015 (PMC5876004; doi:10.3390/metabo8010015)

Supplementary Figure 1. Plasma extraction optimization: a), c), e) Summed sample signal and b), d), f) Average Standard Deviation in relation to respective summed signal for the substance classes amino acids, organic acids and sugars and sugar alcohols for tested extraction methods (A – Isopropanol:Acetone 1:2, B – Methanol, C – Acetonitrile:H_2O, 3:1, D – H_2O:Acetonitrile:Methanol 1+2+2, E – Methanol: H_2O 5:1, F – Acetonitrile, G – Methanol: H_2O, 8:1) (blue – optimized, red – reference method).

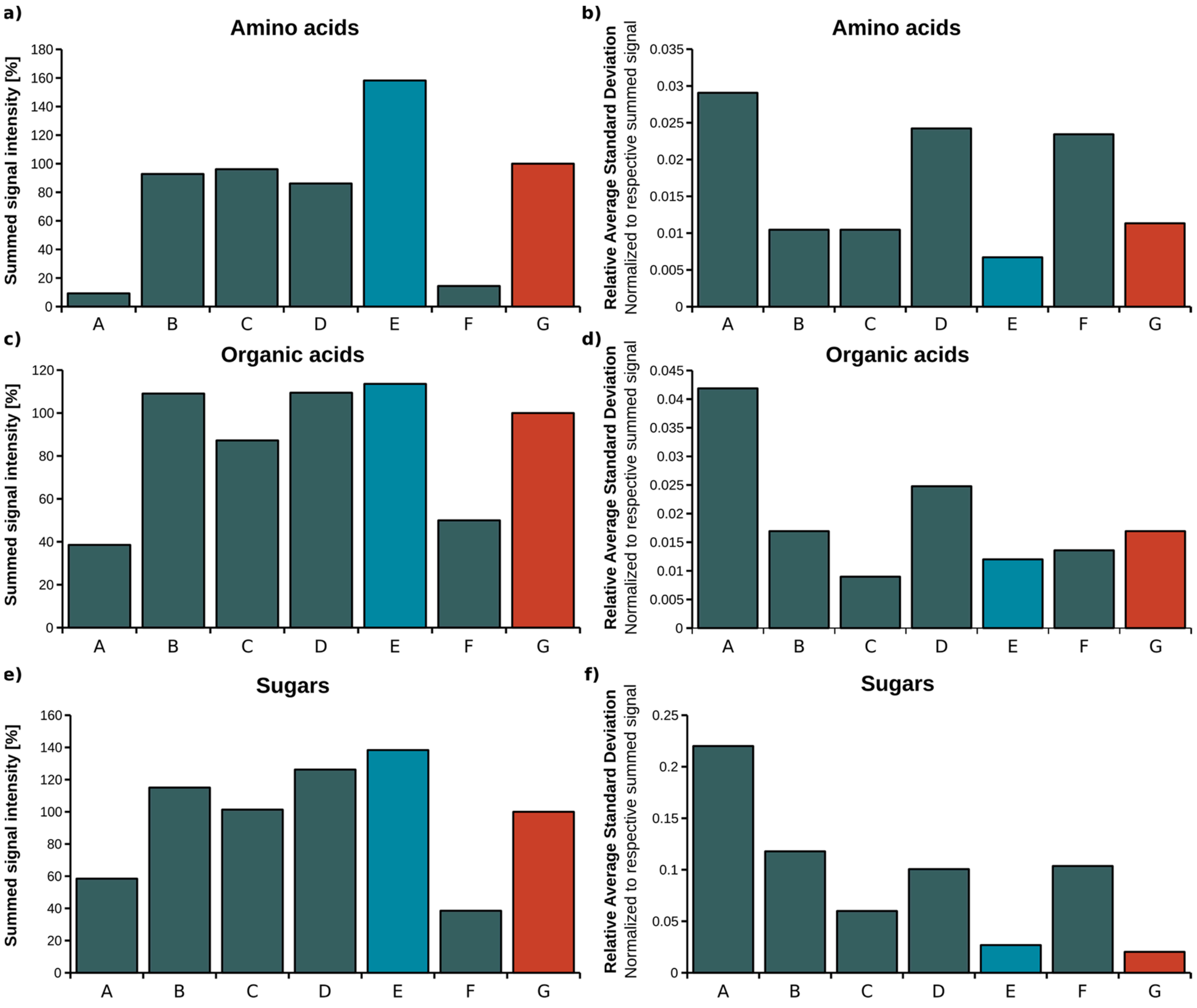

Supplementary Figure 2. Enrichment patterns of additional plasma metabolites of S1, S2 and S3: glycine (M2) and serine (M3) are derived from either starch or protein hydrolysis; glutamine (M5), valine (M5), threonine (M4), isoleucine (M6) and tyrosine (M9) are derived from protein hydrolysis

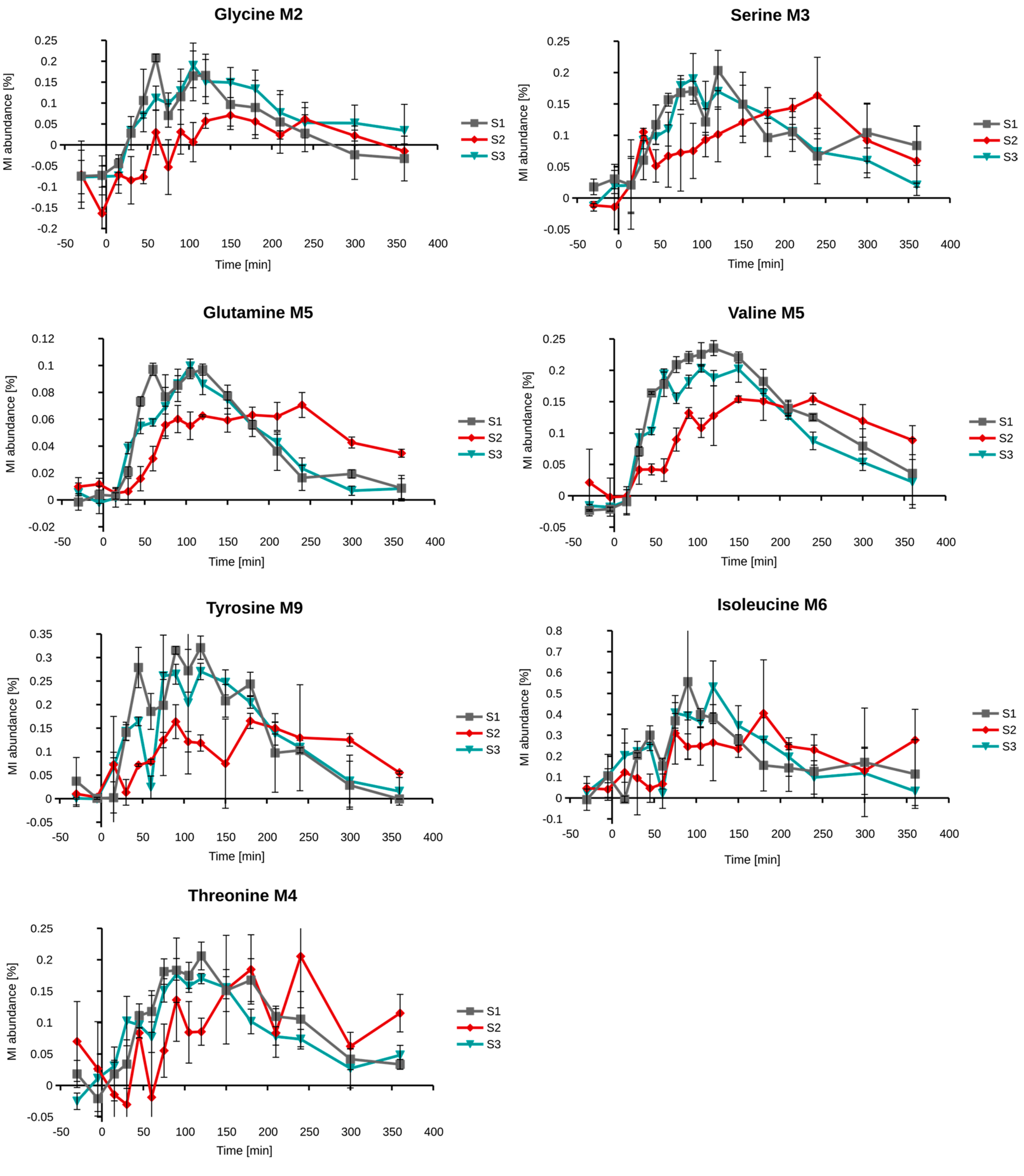

Supplement: Supplementary file 1 [file metabolites-08-00015-s001.pdf]
